# A comparison of preoperative and postoperative testicular volume and blood flow in patients with inguinal hernia, hydrocele, and cord cyst: A prospective cohort study

**DOI:** 10.12669/pjms.332.12487

**Published:** 2017

**Authors:** Ahmet Ali Tuncer, Tamer Peker, Mehtap Berke Acar, Didem Baskin Embleton, Salih Cetinkursun

**Affiliations:** 1 Dr. Ahmet Ali Tuncer, Assistant Professor, Department of Pediatric Surgery, Afyon Kocatepe University, Medical Faculty, Afyonkarahisar, Turkey; 2 Dr. Tamer Peker, MD, Department of Pediatric Surgery, Afyon Kocatepe University, Medical Faculty, Afyonkarahisar, Turkey; 3 Dr. Mehtap Berke Acar, Assistant Professor, Department of Radiology, Department of Pediatric Surgery, Afyon Kocatepe University, Medical Faculty, Afyonkarahisar, Turkey; 4 Dr. Didem Baskin Embleton, Assistant Professor, Afyon Kocatepe University, Medical Faculty, Afyonkarahisar, Turkey; 5 Prof. Dr. Salih Cetinkursun, Afyon Kocatepe University, Medical Faculty, Afyonkarahisar, Turkey

**Keywords:** Children, Doppler ultrasonography, Inguinal hernia, Testis

## Abstract

**Objective:**

To evaluate the effect of inguinal operations performed with a modified Ferguson technique upon testicular volume and blood flow.

**Methods:**

This study involved 23 children receiving surgery for inguinal hernia, hydrocele, and cord cyst. This was a prospective study performed between April 2016 and June 2016 in a medical faculty pediatric surgery unit. The color Doppler ultrasound (CDUS) was used to assess testicular volume and blood flow before and after a modified Ferguson technique surgery. The pre- and post operative testicular volume and blood flow were compared with the contralateral testes. SPSS software was used to statistically analyze the data arising; the Mann-Whitney U test and Friedman test were used to compare samples, and P<0.05 was accepted as statistically significant.

**Results:**

Preoperative and postoperative testicular volumes were not statistically different when compared to contralateral testes. In patients with right sided inguinal pathology, testicular blood flow on the right side was significantly lower than that on the left side (P=0.023). The testicular blood flow was not statistically different compared with the contralateral testes during the first week evaluation and first month evaluation. The blood flow, probably reduced due to the pressure caused by inguinal pathology, was normalized through surgery.

**Conclusions:**

The modified Ferguson technique do not change the testes volume and blood flow.

## INTRODUCTION

Surgery for the repair of inguinal hernia may lead to some adverse long-term effects upon the structures of the inguinal canal, including damage to the testes which might eventually result in testicular atrophy.^1^,^2^ In this study, we aimed to evaluate the short and long term effects of modified Ferguson repair (high ligation) for inguinal hernia, hydrocele or cord cyst upon testicular blood flow, and volume using color Doppler ultrasound (CDUS) in children.

## METHODS

The study was performed prospectively with the approval of the Local Human Ethics Committee. All procedures performed in studies involving human participants were in accordance with the ethical standards of the institutional and/or national research committee and with the 1964 Helsinki declaration and its later amendments or comparable ethical standards. All children included in the study were provided with a patient consent form.

### Study Group

Our analysis included boys who were younger than 17 years and possessing one-sided inguinal hernia, hydrocele, or cord cyst and who were otherwise healthy. The study was performed prospectively between April 2016 and June 2016 in a medical faculty pediatric surgery unit of our university.

### Study Plan

Following diagnosis, patients received a CDUS and testicular volume and blood flow were measured preoperatively. All patients then had surgery with modified Ferguson repair. Testicular volume and blood flow were then reassessed by CDUS on days 7 and 30 following surgery.

### Radiological Evaluation

Radiological evaluation was performed by a single radiologist using CDUS (Hi Vision Preirus, EZU-MT28-S1, and Hitachi) preoperatively and on days 7 and 30 post-surgery. Longitudinal, anteroposterior, and transverse diameters of the testis were measured, and the testicular volume was calculated automatically using the CDUS device (volume = 0.523 × D_1_× D_2_× D_3_, where D_1_, D_2_, and D_3_ were the maximal longitudinal, anteroposterior, and transverse diameters defined by CDUS) for both testicles in each patient.^1^ Peak systolic velocity (PSV) was calculated using the CDUS system (in cm^3^/s) for both testicles in each patient.

### Statistical Analysis

The SPSS 15.0 (Statistical Package for the Social Sciences) software package was used for all statistical analysis. Testicular volumes and blood flow were analyzed as mean ± standard deviation. The Mann–Whitney U test was used to compare differences in testicular volume and blood flow between the tests is receiving surgery and the contralateral testis. Differences in preoperative, early postoperative (7 days post-surgery), and late postoperative (30 days post-surgery) testicular volume and blood flow of each testicle were compared using the Friedman test and P<0.05 was accepted as statistically significant.

## RESULTS

In total, 23 boys were included in the present study. Mean age was 3.59 ± 2.67 years (range: 20 days–12 years). Inguinal pathologies included right inguinal hernia (n=11, 47%), left inguinal hernia (n=7, 31%), right hydrocele (n=2, 0.8%), left hydrocele (n=2, 0.8%), and right cord cyst (n=2, 0.8%). All patients received modified Ferguson repair. In addition, partial hydrocelectomy was performed in hydrocele patients, and cyst excision was performed in cord cyst patients. All patients had been followed up for one month post-surgery; there were no complications related to the surgery and no recurrence. There were no statistically significant differences in terms of preoperative, early postoperative or late postoperative volume in repaired testes compared with the contralateral testes (P<0.05; Table-I).

**Table-I:** Statistical analysis of preoperative, early postoperative and late postoperative testicular volumes compared with contralateral testes.

*Operation side*		*Right testis: Preoperative volume*	*Right testis: Postoperative 7th day volume*	*Right testis: Postoperative 30th day volume*	*Left testis: Preoperative volume*	*Left testis: Postoperative 7th day volume*	*Left testis: postoperative 30th day volume*
Right	N	16	16	16	16	16	16
	Mean	0.6963	0.7138	0.6738	0.6000	0.6263	0.5988
	Std. Deviation	0.22553	0.27136	0.18629	0.18316	0.20529	0.13720
Left	N	7	7	7	7	7	7
	Mean	0.6814	0.6129	0.5700	0.6143	0.6114	0.6171
	Std. Deviation	0.26674	0.20254	0.22657	0.27397	0.28299	0.31742
Total	N	23	23	23	23	23	23
	Mean	0.6917	0.6830	0.6422	0.6043	0.6217	0.6043
	Std. Deviation	0.23267	0.25229	0.20011	0.20830	0.22500	0.20097
	P	0.789	0.285	0.192	0.815	0.503	0.462

Preoperative testicular blood flow in the right testicle was significantly reduced in patients with right-sided pathologies compared to the left testicle (P=0.023) (Table-II). In contrast, there was no statistically significant difference in postoperative early and late testicular blood flow when right and left testicles were compared (P >0.05 for all groups) (Table-II).

**Table-II:** Statistical analysis of preoperative, early postoperative and late postoperative testicular blood flow compared with contralateral testes.

*Operation side*		*Right testis: preoperative volume*	*Right testis: posoperative 7thday volume*	*Right testis: postoperative 30th day volume*	*Left testis: preoperative volume*	*Left testis: postoperative 7th day volume*	*Left testis: postoperative 30th day volume*
Right	N	16	16	16	16	16	16
	Mean	7.4688	8.8875	7.4813	7.4063	8.0688	7.3750
	Std. Deviation	1.67679	2.83170	2.34072	1.93096	2.30441	2.25137
Left	N	7	7	7	7	7	7
	Mean	9.9286	9.0000	8.9000	8.3571	8.9571	8.8286
	Std. Deviation	2.94036	2.41454	2.19773	2.45279	1.71936	2.98145
Total	N	23	23	23	23	23	23
	Mean	8.2174	8.9217	7.9130	7.6957	8.3391	7.8174
	Std. Deviation	2.36944	2.65706	2.34488	2.09360	2.14513	2.51950
		P	0.023	1.000	0.116	0.192	0.332	0.204

When preoperative, early postoperative, and late postoperative testicular volumes and blood flow of the testes with a pathological condition were compared using the Friedman test, there was no statistically significant difference in testicular blood flow (P >0.05 in all groups). There was a slight increase in testicular blood flow in the pathological testes during the early postoperative period that returned to normal during subsequent postoperative measurements; however, this difference was not statistically significant (Table-III).

**Table-III:** Preoperative, early, and late postoperative comparisons of testicular volume and testis blood flow on testes receiving surgery.

*Groups*	*Mean rank*	*P value*
***Volume of the right testicle***		0.661
Preoperative	2.11 cm^3^	
7^th^day	2.02 cm^3^	
30^th^day	1.8 cm^3^
***Volume of the left testicle***		0.795
Preoperative	2.11 cm^3^	
7^th^day	1.96 cm^3^	
30^th^day	1.93 cm^3^
***Blood flow of the right testicle***		
Preoperative	1.98 cm^3^/sn	0.503
7^th^day	2.17 cm^3^/sn	
30^th^day	1.85 cm^3^/sn	
***Blood flow of the left testicle***		0.182
Preoperative	1.83 cm^3^/sn	
7^th^day	2.30 cm^3^/sn	
30^th^day	1.87 cm^3^/sn

Since diastolic blood flow could not be measured in this study, it was not possible to calculate the resistive index. However, several studies have noted that diastolic blood flow may not be calculated accurately in prepubertal patients.^3^,^4^

## DISCUSSION

Several studies have been conducted to assess testicular damage following inguinal surgery (Table-IV) to determine the presence and causes of testicular damage and develop appropriate measures to preserve the fertility of the patients involved.^5^-^7^ These studies focused upon preoperative and postoperative testicular volume and blood flow and spermiogram of the patients in relation to the techniques used for inguinal surgery.^8^,^9^ The most frequently used factors for this purpose involve calculating testicular volume, blood flow indices of centripetal and capsular arteries including PSV and end-diastolic velocity (EDV), and resistivity index (RI) with grey scale and CDUS.^10^ Schier et al. also reported the use of a neuromonitoring device (which combines light spectroscopy and the laser Doppler technique) to evaluate real-time hypoxia.^11^ In adult patients, changes in spermiograms are also monitored, although such assessments are not possible in prepubertal boys.^12^

**Table-IV:** The effects of inguinal operation techniques upon testicles: a review of the literature concerning childhood studies.

*Study*	*No. of patients*	*Operation technique*	*Evaluation technique*	*Follow-up*	*Result*
Palabiyik et al.	51	High ligation	CDUS	Preoperative Postoperative 7^th^day Postoperative 30^th^day	Transient changes in testis vascularization in early postoperative period but turns to normal late postoperatively.
Parelkar et al.	112	Laparoscopic ring closure	CDUS	Preoperative Postoperative 48 hours Postoperative 6 months	NS
Celebi et al.	72	High ligation Schier Montupet	CDUS	First week- first month	NS
Leung et al.	173	High ligation	US- testis volume	6 to 123 months	NS
Schier et al.	65	Laparoscopic internal ring closure technique	Neuromonitoring device (which combines light spectroscopy and laser Doppler technique)	Before and after anesthesia, Before and after surgery, Postop 6 weeks	NS

CDUS:Color Doppler ultrasound, US:Ultrasound, NS:Not specific

In our present study, we used CDUS to determine testicular blood flow and testicular volume measurements. This is a widely used non-invasive technique that is commonly used to evaluate both the structures in the inguinal region, and the perfusion of the testes. RI is also a valuable measurement, but for this, it is necessary to determine diastolic blood. However, the measurement of diastolic blood flow or EDV may not be possible in prepubertal children, and as a consequence, RI was not evaluated in our study.^4^

Whereas high ligation, modified versions of the Ferguson and Mitchel Banks technique, is the most frequently used repair technique for children, surgeons usually prefer tension-free techniques using prosthetic matches for adults. Laparoscopic repair is also becoming an increasingly popular approach.^11^,^13^

A literature search carried out by Dilek found that there was no difference in testicular blood flow after open or laparoscopic inguinal hernia surgery.^14^ However, Gurbulak et al. found that although all parameters (PSV, EDV, and RI) were affected in the early postoperative period, they all returned to initial levels during the late postoperative period.^5^ Another study by Palabiyik et al. did not find any difference in testicular volume in the preoperative, early, and late postoperative periods in their study of 51 boys but did identify statistically significant differences in the preoperative and early postoperative values of peak systolic flow and RI. These differences were associated with postoperative vasoconstriction due to pain and edema.^6^ These previous findings were consistent with those of our current study except that while the peak systolic volume was increased during the early postoperative period, this difference was not statistically significant.

Laparoscopic hernia repair has become increasingly popular over recent years. Celebi et al. performed a study to evaluate the results of laparoscopic Schier and Montupet inguinal hernia repair and open inguinal hernia repair in 72 children older than two years with single-sided inguinal hernias. Early postoperative RI and PSAF measurements were higher than preoperative values, although this difference was not statistically significant.^15^ The findings of this previous study concurred with those of our present study.

Schier et al evaluated 65 further cases who had laparoscopic hernia repair. In this instance, combined light spectroscopy and laser Doppler (neuromonitoring device) were used to reveal that there were no differences in terms of testicular perfusion. This technique is thought to be superior to CDUS since it can also reveal local hypoxia.^11^ One of the limitations of our current study is that we were unable to evaluate the small arteries that play a key role in testicular perfusion.

In another study, Parelkar et al. used CDUS to evaluate 100 patients receiving laparoscopic hernia repair preoperatively, and in early (2 days) and late (6 months) postoperative periods but did not find any significant changes in testicular perfusion.^16^ Of note was the fact that RI could not be evaluated in 25% of the cases in this previous study, thus clearly demonstrating the problems encountered measuring this parameter.

In the current study, we found that there was a statistically significant reduction in testicular blood flow in the right testes in patients with pathologies on the right-side (p=0.023). This difference disappeared postoperatively. It was thought this was caused by the pressure of the hernia, hydrocele or cyst upon local blood vessels. Similar results were reported by Aso et al.^17^ It is possible, however, that the statistical insignificance of the blood flow changes on the left side may be because of the small number of left-sided cases (n=7).

In a large-scale study involving 8500 patients with infertility attending infertility clinics, Yavetz et al. identified 565 (6.65%) with prior inguinal hernia repair with or without testicular atrophy. This is a much higher value than those reported previously, which were in the order of 0-2%.^18^ Incidentally, the authors of this previous study also observed high values of follicle stimulating hormone (FSH) and proposed that testicular damage may represent the result of ischemic orchitis or immunological reactions.^18^

It is evident that these earlier findings in adult patients contradict the findings of those from children that generally failed to identify testicular damage following inguinal hernia operations.^15^,^16^ It appears that it is necessary to follow children who have received inguinal hernia operations for long periods, potentially by annual examination including CDUS evaluation of the testes. The testis requires a stable blood flow to function effectively. If testicular blood flow decreases or RI increases, then testicular ischemia and damage may occur. Pinggera et al. used CDUS to examine 320 testes in 160 patients, in which 80 had a normal spermiogram and 80 had a pathological spermiogram.^7^ While testicular volumes were similar when compared between the two groups of patients, there was a significantly higher RI in patients with pathological spermiograms. These authors proposed that in adult males, a RI higher than 0.6 may be predictive of a pathological sperm count and could therefore be used in routine clinical practice to safely diagnose subfertile males.^7^

In conclusion, our study has demonstrated that open inguinal surgery in boys did not affect testicular volume or blood flow. On the contrary, surgery increased blood flow, which was shown to be reduced preoperatively. These findings contradict with the high proportion of infertile males who had undergone inguinal hernia surgery earlier in their lives. Although other publications report the negative effects of inguinal hernia surgery upon male patients, these usually involve inguinal hernia surgeries carried out during adulthood.^18^ We recommend that the long-term effects of childhood inguinal hernia surgery upon fertility should be evaluated separately.

### Authors` Contribution

**AAT, SC** conceived, designed and did statistical analysis & editing of manuscript.

**AAT, TP, SC, MBA, DBE** did data collection and manuscript writing.

**SC** did review and final approval of manuscript.
